# Measuring the Meissner effect at megabar pressures

**DOI:** 10.1093/nsr/nwz094

**Published:** 2019-07-17

**Authors:** Dmitrii Semenok, Artem R Oganov

**Affiliations:** Center for Energy Science and Technology, Skolkovo Institute of Science and Technology, Russia

Since 2014, when the research group of Professor Tian Cui (Jilin University) predicted [1] (and experiments [2] confirmed) the existence of an unusual high-pressure compound, H_3_S, with superconductivity at 191–204 K, a new era in studies of superconductivity began. In 2019, a new record of high-temperature superconductivity was set, with LaH_10_ experimentally proven to be a superconductor with a nearly room-*T*_c_ of 250–260 K [3,4]. H_3_S and LaH_10_ cannot be used in practical applications, because they exist only at megabar pressures, but their study may hint at which compounds can be room-temperature superconductors at normal pressure. The unusually high electron–phonon coupling constants (λ > 2) of these materials also make them interesting from the physical point of view. 

The first test of superconductivity under pressure is the measurement of electrical resistivity and of the isotope effect. However, magnetic measurements are also highly desirable. From the technical point of view, such experiments are still extremely non-trivial. The problem is to achieve good signal/noise ratios in measurements of extremely small values of the magnetic flux change—Ф' = S·*dB/dt* and induced potential difference (~ 10–100 nV) arising when the external magnetic field is pushed out of the superconducting sample (~ 10^−5^ mm^3^ volume) compressed in a diamond anvil cell (DAC) [5].

**Figure 1:**
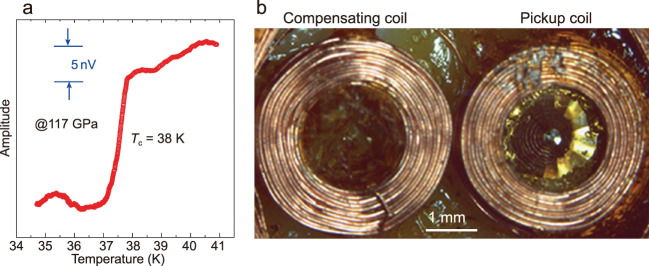
(a) Magnetic susceptibility signals of compressed H_2_S at 117 GPa. (b) A pickup coil wound around a diamond anvil and a compensating coil connected in opposition [6].

In a recent paper published in *National Science Review* [6], the group of Tian Cui studied magnetic transitions in compressed sulfur hydride at ultrahigh pressure [3]. This report closes the gap in previous experimental studies of the Meissner effect in H_3_S and identifies the superconductivity of H_x_S compounds employing an *in situ* alternating-current magnetic susceptibility technique at pressures over 1 Mbar. They determined the *T*_c_(*P*) dependence in pressure ranges 117–130 and 149–175 GPa, confirming the previous results on H_3_S and pointing to the formation of additional phases with stoichiometries other than H_3_S (see [7] for a study of other H-S compounds) and lower *T*_c_ < 100 K (Fig. 1). The work of Cui's group provides a new insight into the superconductivity of hydrides and sets a new standard for experimental studies of superconductivity at high pressure.
